# Cultivating the conditions for care: it's all about trust

**DOI:** 10.3389/frhs.2024.1471183

**Published:** 2024-12-09

**Authors:** Allison Kooijman, Carolyn Canfield

**Affiliations:** ^1^ University of British Columbia, Okanagan Campus, Kelowna, BC, Canada; ^2^ University of British Columbia, Vancouver, BC, Canada

**Keywords:** patient safety, patient engagement, trust, cultivating care, restorative approach

## Abstract

This perspective article shares the viewpoints of two long-standing patient safety advocates who have participated first-hand in the evolution of patient engagement in healthcare quality and safety. Their involvement is motivated by a rejection of the common cruelty of institutional betrayal that compounds harm when patient safety fails. The advocates have sought to understand how it can be that fractured trust spreads so predictably after harm, just when it most needs strengthening. Instead, the abandonment of trust upends healthcare values and effectiveness at interpersonal, systemic and structural levels. They argue that authentic care (healthcare that is truly caring) transcends mere service delivery, thus embodying an inviolable commitment to mutual well-being, compassion and generosity. The advocates identify the influence of social determinants, such as culture, identity, and socioeconomic status, as critical to trust formation, where pathogenic vulnerability exacerbates existing inequalities and further impedes trust. The advocates call for a shift from transactional to relational, trust-based interactions that explore the potential for mobilizing restorative justice principles to repair harm and rebuild trust, enabling dialogue, mutual understanding and systemic improvement. Trust, they assert, is born in relationships, not transactions. The bureaucratic, legal and resource constraints that often impair meaningful interactions, also cause moral distress to healthcare providers and poor care quality for patients. They argue that central to the current healthcare crisis is the fundamental need for genuine connection and trust, framing this as both a practical necessity and a confirmation of humanity as intrinsic to healthcare. The advocates envision a future where patient engagement is integral to patient safety to prioritize epistemic justice, mutual respect and compassionate care, to restore healthcare as a cohesive, supportive and deeply human endeavor. They query what contributions a restorative approach could make to centre trust as necessary for cultivating the conditions for care in our healthcare system.

## Introduction

We are grateful, as patient advocates, to have been asked to contribute to this special edition of Frontiers in Health Services: *The Future of Patient and Family Engagement in Quality and Patient Safety*. At first glance, the task of articulating what it is that we choose to do—the essence of our daily activism, what we eat, sleep, and breathe—might seem straightforward. Yet, we find it to be unexpectedly challenging. Perhaps it is because what we see to be most crucial in the healthcare space does not show up in the accounting: we do not measure it. Relational trust lies outside the balance sheet as a frivolous externality, if considered at all.

Our commitment to ensuring the patient voice is alive and well in quality and patient safety initiatives springs from the very depths of our experiences of betrayal, exclusion and fractured trust. It is anchored in profound pain and animated by unwavering hope. Our work and contributions are born of personal transformative experiences within the healthcare system, and driven by far more than the desire to improve; they arise from a relentless allegiance to trust and mutually compassionate caring. If you know either of us, you will know that our conversations often revolve around the need to cultivate the conditions necessary for authentic care to flourish—a care that transcends the mere delivery of services and touches the essence of what the “caring” in healthcare truly should be: an intensely human exchange for mutual well-being, compassion, generosity and trust.

To say we are passionate about patient engagement and safety barely captures the full spectrum of our emotions, as they are grounded in complex narratives, overlaid with years of frustration, determination and sorrow, with a perpetual optimism entwining our persistence. Our dedication is not meeting a professional competency, but rather a moral response to harm's call to action, as witness and victim—a need to rectify, to heal, and to elevate, driven by our own narratives of loss, hope, and resilience. Our vision is not just about changes to systems and policies; it is about lives lived and lost, real suffering, and the stubborn belief that things can, and should, be better. We believe that involving those with lived experience in reform really can be transformative for the harmed and for those accountable.

When prodded to dream about “the future of patient engagement in patient safety,” we see a need to liberate activism from cold co-design jargon and recognize it for what it is—humanity—a thorough and careful sharing of truth between patients and those involved with healthcare improvement. This ‘engagement’ cannot be relegated to a mechanical listing of needs to be matched to services, the ticking of boxes. Rather it must nurture tentative relationships, eventually to blossom into aligned understanding, to fortify mutual respect and aid collaboration, to relax power gradients, and to emerge as allyship, even friendship. We know this because we have experienced partnerships built on mutual desires to move mountains, together. The future of patient engagement in patient safety could nurture this type of synergy to protect an open-hearted dance to evolve, to grow more intuitive and habitual, and to remain closely attuned to relational dynamics at its very core. We want to recognize a powerful creative honesty within the domain of patient engagement, when we embrace true partnership more deliberately. We want to tell you that we have found healing only where we could find trust, and then only through deeply invested generosity from within the system. We want to emphasize the centrality of cultivating and sustaining trust as a focal area for progress in the quality and patient safety space.

## The social determinants of trust

A deep dive into the fundamental issues in our healthcare system means grappling with how we depersonalize, institutionalize and codify the most powerful and shattering human emotions where humanity intersects with illness, suffering, fear and mortality. Spanning the breadth of harm in health care invariably confronts life and death, also living with unexpected profound disability. By its very nature, harm tugs at healthcare's roots in trust. In the domain of healthcare, trust is not merely an operational asset, but the fertility from which all forms of genuine caring arise. Healthcare's dimensions of trust transcend the confines of professionalism and frame the sanctuary where mutual respect, safety and vulnerability stir. Within such a sanctuary, patients and providers may each find enough mutual recognition to lower their guard. This is where care happens.

We have some ideas about trust, the nature of trust, and what it demands. We believe trust underpins healthcare at interpersonal, system, and structural levels. We need to know that our healthcare providers trust sufficiently in themselves to hold our vulnerability, as well. We need to be able to trust each other as patient and provider, as we agree on the direction to travel through challenges we encounter together. We need to be able to trust that our institutions and policies are capable of confirming the trust of our healthcare providers, as they stretch their own vulnerability to represent the mandates of their employer. In this way, the exchange of trust and vulnerability becomes a reciprocal pair, equally authenticating emotional investment across teams, and even between society and those who stand publicly accountable for healthcare services.

Our willingness and ability to trust healthcare professionals and their institutions are affected by our upbringing, culture, racial identity, age, gender, education and economic status. Our willingness and capacity to trust healthcare are also affected by personal histories of illness and care, and vicariously by those of family, friends, and community. Vulnerability encompasses our worries over susceptibility to illness and suffering, our anticipation of rapid or intractable death, but also the social constructs of pathogenic vulnerability (1) that result from unequal or discriminatory social, political, economic arrangements and their aggravation, or their exacerbation. Trust provides a foundation for honest care where empathy and generosity circulate between patient and provider, transforming timed exchanges of information into moments of potent meaning-making.

The journey to embedding this level of trust across the healthcare spectrum is fraught with structural and cultural barriers. Bureaucratized healthcare strips interactions of their humanity, trading anchoring human exchanges for perfunctory transactions, marked by dizzying paperwork and protocol. Trust is born in relationship, not transaction. The shadow of legal ramifications casts a chill, corrosive fear of “the other”, inhibiting open communication for fear of litigation, which in turn drains the trust that is critical for transparent and generous dialogue to support care. Constant pressure on resources means that even the most dedicated providers may find themselves unable to deliver the level of care they aspire to. Under such constraints, the potential for meaningful interactions is impaired and diminished, leading to a cycle where trust is eroded, moral distress becomes the reality, and the requisite motivation to provide empathetic care is undermined (2). Navigating these challenges requires an intentional and conscientious effort to cultivate the conditions where trust can flourish. Reforming healthcare priorities and practices requires enhancing the capacity for empathy, promoting transparency, and fostering a collaborative atmosphere that welcomes contributions from both patients and providers.

On reflection, we have pondered whether it is too idealistic, or unhelpful, to speak of the need to deepen trust during a time of healthcare crisis; we are convinced that there is no better time. The current challenges only underscore the need for genuine human connection—a connection that has been eroded by the drive for transactional efficiencies which proliferate within the industrial healthcare system. Such interactions are commonly devoid of mindful presence, touch that once defined patient care and vested meaning for patient and provider alike. In a crisis, our call to elevate trust and to create conspicuous conditions for genuine care is not just about aspiring to an ideal. We advocate a thoughtful assertion of trust as the very essence of care and what it is to be human. We are constituted in and through our relationships with others (3) as inherently social beings; the integrity of our relational connections can increase our wellbeing, or cause harm. Our message is a reminder that healthcare, at its core, is about people caring for people, about meeting human needs with compassion and competence. The crisis we are truly facing is as much about restoring this fundamental truth about who we are as living, breathing and feeling beings, as it is about addressing the logistical and medical challenges at hand. Jointly, this is a crisis of trust and a crisis of care.

This emphasis on connection and trust is not a luxury but a necessity, critical to healing not only individual patients, but healing the healthcare system as a whole. In moments of crisis, the instinctive human response should be to come together, to support, and to understand—principles that are also pillars for the provision of quality patient care. By reinvigorating these principles, we can transform the landscape of healthcare from one that is fragmented and impersonal, to one that is cohesive and deeply human. In this sense, speaking pragmatically about trust and advocating for deeper connections in times of crisis, is perhaps the most grounded and practical approach we can take. It is an approach that attends to the complexities of human health, the limitations of medical knowledge, the vulnerabilities of bureaucratic systems, and the profound potential of human relationships. By shifting our focus to these accommodations, we are not just navigating a crisis but reshaping the future of healthcare culture into one that truly comes home to honour its purpose—to care, to heal, and to safeguard the well-being of the community it serves. We have lost our way in healthcare, but we have the capacity to reconnect.

This process of reconnection involves coming to grips with the course of events that have led to the current state of disconnection. The evolution of patient safety—from an early focus on professional dominance, to the more recent emphasis on systemic complexity and patient-centered care—has shaped our understanding of agency and influence. Initially, the field of healthcare viewed harms and errors through a lens on individual fallibility, responsibility and culpability. Over time, this perspective shifted towards viewing errors as preventable, but often the result of inherent system faults, allowing individual missteps and cascading failure. Increasingly the goal of “harm-free healthcare” has faded (4) as safety science has shifted from error prevention to understanding safety in complex systems (5). This shift has been instrumental in revealing how relationships of trust can animate healthcare systems, when we recognize shared responsibility for embracing complexity and the unpredictability of our healthcare system (6). As we examine the role of trust, it is essential to acknowledge how the evolution of patient safety influences and challenges current strategies, contexts and culture.

## Why a restorative approach can help with our “trust” problem

Referencing *justice* in the context of patient safety has been often met with alarm by healthcare providers, eliciting fear, perhaps rightfully so when we have seen a default response seemly driven by litigation terror. This has been a challenging area of exploration for us when thinking through the needs of all parties caught in the throes of uncertainty when healthcare does go badly. While justice notions of fairness, transparency, moral action and epistemic justice (7) may be exactly what is necessary in this context, our legal structures evoke a justice that is less relational and more retributive. A new literature is reaching into the legal frameworks and orientations of healthcare, to report on outputs of alternative legal theories and positions (8). During our time in the safety realm, efforts to implement a *just culture* in healthcare have dwindled as we have collectively come to reckoning that it is not possible to have a just culture in a retributive system (9). Our experiences mirror the findings from Wailling et al., where responses to adverse events in our healthcare system are observed as often serving to compound the experience of harm for both patients and healthcare providers (10). Resonant to our own experiences of patient harm, Ray Nickson and Alice Neikirk relate their experience in learning about traditional responses to healthcare harm (11):

The system that investigates and responds to medical negligence, we would learn, was not about justice. It was not about avoiding repetition of mistakes. It was certainly not about healing. It was not even about punishment. What we would have benefitted from was a process that revealed truth and encouraged dialogue between us, the hospital and the health professionals—a process that would have allowed us to hear, from the medical staff, a frank and honest narration of what had happened. We would have benefitted from a process that allowed us to express our pain and grief and to share how the actions of the healthcare professionals affected us. We wanted acknowledgment of those harms from the responsible parties. We wanted to be part of a process that helped doctors and families in the future to avoid the harms that we had experienced.

The time has arrived when the demand for social justice reverberates with clarity and urgency. Our institutions, and traditional notions of justice as punishment are not aligned or intended to delivery on the type of justice that is being requested. What is sought is a healing justice with quality and equality of relationship at its core. This is a justice where people are seen and heard as though they matter, and where the context and intersections in which they experience their reality, count. This is a justice where human fallibility is acknowledged as fact, and where examinng the past to learn, and working together to chart the best path forward will mean moving away from punishment, just as surely as away from “blame and shame”. This view of justice is championed in the work of feminist relational legal scholar, Jennifer Llewellyn, who theorizes restorative justice as a relational theory of justice, grounded in a commitment to understanding “the fact of relationship and connection as central to the work of justice” (12, p. 89). Llewellyn further argues:

Relational theory, thus, has significant implications for our thinking about justice. But it profoundly affects not only our thinking but also our approach to doing justice. Indeed, it requires an adjustment in the very way that we understand the work that justice requires. Taking relationship as the focal point of justice requires a contextual approach. The question of what justice requires, then, cannot be met by standard and formulaic answers but, rather, must take into account what is needed in a particular context to achieve just relationships between and among the parties involved (p. 98).

Furthermore, conversations within patient groups in quality and patient safety are moving towards increasingly sophisticated thinking around re-orienting towards a fresh perspective of systems and understanding of justice. This emerges from recognition of the system-centered environment in which healthcare providers are regulated and do their work: stressed systems which fail them too (13). We see great potential for a generosity of spirit and openness to listen and learn to create the allyship and space in which we can walk together in our collective effort to co-create the conditions in which trust and care will flourish.

## Conclusion

Our journey as patient advocates underscores the transformative potential of trust and our view that a restorative approach to addressing the deep-seated lapses within our healthcare systems is necessary to humanize healthcare systems. Trust is not merely an operational asset; it is the generosity from which all authentic care arises. It is born in relationships, not transactions, and its erosion has had far-reaching consequences for both patients and providers. A restorative approach offers a relational and trauma-informed theoretical framework that shifts from assigning blame, to bringing understanding to the multifaceted impacts of harm on well-being. By fostering empathetic and respectful dialogue, restorative practices create conditions for psychological safety, allowing for the repair of broken connections and the validation of lived experiences. This approach invites all affected parties, ensuring every perspective can shape the understanding of what happened, and then guide the actions required for healing and learning.

The current healthcare crisis, marked by alienation and exhaustion from reliance on transactional exchanges, underscores the necessary urgency in reinvigorating foundational principles of trust and empathy. By embracing a restorative approach, we can remodel the healthcare landscape into one that is nurturing, cohesive and deeply human. This involves not only addressing the immediate needs arising from adverse events, but also embedding restorative values and principles within policies, governance structures, and organizational cultures. To achieve this, we must engage in authentic partnerships and consider cultural diversity, particularly the wisdom and practices of Indigenous communities. Policies must be co-created with all those affected, guided by restorative principles that honour inclusive dignity and respect. Building these perspectives into the design and evaluation will provide a protective buffer when harm in healthcare inevitably occurs, and will establish a system to flourish where learning and healing travel hand-in-hand.

Ultimately, the goal is to create a healthcare environment that honors the dignity and worth of every individual, fostering genuine care and enhancing both the quality of care and the quality of life for all involved. By taking a restorative approach, we cultivate the conditions necessary for genuine care and renewed trust in our healthcare system, allowing it to fulfill its purpose—to care, to heal, and to safeguard the well-being of the community it serves.

## Data Availability

The original contributions presented in the study are included in the article/Supplementary Material, further inquiries can be directed to the corresponding author.
